# Modified extracorporeal septoplasty: prospective study

**DOI:** 10.1016/j.bjorl.2024.101398

**Published:** 2024-02-22

**Authors:** Raphaella de Oliveira Migliavacca, Michelle Lavinsky, Olívia Egger de Souza, Eduardo Priesnitz Friedrich, Otávio Augusto Gonçalves Dias Cionek, Leonardo Ferreira Subda, Bárbara Luiza Bernardi

**Affiliations:** a Hospital de Clínicas de Porto Alegre, Porto Alegre, RS, Brazil; b Universidade Federal do Rio Grande do Sul, Faculdade de Medicina, Departamento de Otorrinolaringologia, Programa de Pós-Graduação em Ciências Médicas: Otorrinolaringologia, Porto Alegre, RS, Brazil; c Programa de Pós-Graduação em Ciências Pneumológicas, Porto Alegre, RS, Brazil

**Keywords:** Modified extracorporeal septoplasty, Keystone area, Nasal obstruction, NOSE, ROE

## Abstract

**Objectives:**

This study aimed to evaluate quality-of-life and satisfaction outcomes in patients undergoing the MES using the Portuguese version of the Nasal Obstruction Symptom Evaluation (NOSE-p) and Rhinoplasty Outcome Evaluation (ROE), and also to evaluate the frequency of possible complications of this technique.

**Methods:**

We conducted a single-center prospective study with patients who had the indication for MES, from May 2016 to September 2020 at the Facial Plastic Surgery Clinic of Otolaryngology Department of the Hospital de Clinicas de Porto Alegre. The primary outcome was the relative postoperative change in NOSE-p. Secondary outcome was the variation in ROE, a validated quality-of-life questionnaire for rhinoplasty patients.

**Results:**

Of the 31 patients submitted to extracorporeal septorhinoplasty who were evaluated, twenty-seven patients were included. Preoperative and postoperative NOSE-p scale scores were 65.2 ± 29.9 and 23.5 ± 26.7, respectively (mean differences of 42.04; [95% CI 27.35–56.73]; *p* <  0.0001). Pre and postoperative ROE scores were 38.3 ± 24.3 vs. 67.29 ± 29.7, respectively (mean differences of −29.02; [95% CI −40.5 to −17.5]; *p* =  0.0001). Residual septal deviation was verified in 2 patients (7.4%).

**Conclusion:**

Most of the patients submitted to modified extracorporeal septoplasty had a significant improvement in quality of life scores of nasal obstruction, with good aesthetical outcomes and low indices of postoperative complications.

**Level of evidence:**

Level 3.

## Highlights

• Keystone preservation reduces the risk of destabilization of the nasal dorsum.
• Modified extracorporeal septoplasty improved quality of life and aesthetics.
• Low index of complications after Modified Extracorporeal Septoplasty.

## Introduction

Nasal obstruction is one of the most prevalent complaints in otorhinolaryngology since approximately one third of the population has some degree of nasal obstruction, and one quarter of these patients pursue surgical treatment.^1^ Septal deformities are present in up to 77%–90% of the general population.^2^, ^3^ In addition to that, septoplasty is one of the most common surgeries performed by otolaryngologists.^4^ This procedure is indicated to treat nasal obstruction caused by a deviated nasal septum and it is also an essential step in rhinoseptoplasty in order to harvest graft material and obtain optimal aesthetical and functional results.^4^, ^5^

The nasal septum is crucial to nasal support and to adequate nasal function. In patients with nasal obstruction and mild to moderate deviated septum, most cases can be treated with the conventional excision septoplasty technique, especially if the deviation is localized with spurs or like a septal tilt deformity.^5^

On the other hand, the treatment of a severely deviated septum is often a challenge, particularly in those deviations located at its anterocaudal and dorsal portions, demanding different surgical techniques for its correction. Guyuron described a classification system for septal deviations subdividing them into 6 different types of anatomic deformity. C-shaped or S-shaped anterior-posterior septal deviations may lead to deformities of the dorsal L strut, while C-shaped or S-shaped cephalocaudal deviations can lead to deformities of the caudal (anterior) L-strut. The remaining 2 classifications do not involve the L strut.^6^, ^7^ Unfortunately, complex deformities involving the L-strut are often underappreciated. Most and Rudy emphasized two major challenges of anterocaudal deviations: the association of internal nasal valve narrowing and aesthetic deformities like irregularities of dorsum and columella and nasal tip ptosis.^8^

Some L-strut deformities can be treated effectively with traditional non-extracorporeal techniques. The most commonly used in situ techniques on this purpose are swinging door, septal translocation, cartilage scoring, bony batten grafting, septal extension grafting and other excision and suturing methods.^9^, ^10^, ^11^, ^12^, ^13^, ^14^

However, the extracorporeal septal reconstruction, when properly executed, can eliminate virtually any L-strut deformity, and the extracorporeal technique remains the gold standard for severe anatomic derangements of the septal L strut.^6^

It has been a long way since the first description of extracorporeal septoplasty by King and Ashley in 1951^15^ and the consolidation of this technique in early 1990’s, especially with a consistent series of cases published by Gubish et al. in the past 30 years.^16^, ^17^, ^18^, ^19^, ^20^ During this time, extracorporeal septoplasty technique and its variations have shown to be extremely reliable methods to treat complex septal deviation, particularly those deformities involving the L-strut, performing low complication rates and optimal aesthetic and functional results, as showed in most studies published since then.^5^, ^6^, ^21^, ^22^

The Modified Extracorporeal Septoplasty technique (MES) described by Most and many other authors, with preservation of the keystone area, is an attractive variation of the conventional extracorporeal technique. That is because MES prevents destabilization caused by resection in the keystone area and its underlying risk of a saddle nose deformity or other dorsal irregularities, ultimately avoiding the necessity of implementing more aggressive and time demanding maneuvers to preserve the stability (percutaneous suture, suture to the ethmoid bone, nasal bone drilling) and revision surgery.^23^, ^24^

Furthermore, Wilson and Mobley^25^ described a simplification for the fixation of the anterior portion of the neoseptum. They replace the need of drilling a hole into the nasal spine and suturing the neo septum to the maxillary crest to achieve stable midline fixation. To accomplish that, the neoseptum fabricated must be longer than the original reconstructed nasal septum thus its anterior fixation is made by simply extending the caudal neoseptum into the medial crura of the inferior lateral cartilages and using a tongue-in-groove like suture.

In the last few years, for complex septal deformities involving the L-strut, we use a sum of those modified maneuvers which preserve the philosophy of extracorporeal septoplasty, as described by Gubish, but makes the surgery less technically challenging, decreases the surgical time, eliminates the need of drills, and turn the procedure more reproductive so nasal surgeons can embrace an effective method to correct most of the complex septal deformities.

This study aimed to evaluate quality-of-life and satisfaction outcomes in patients undergoing the MES technique, implemented in our practice in a tertiary care institution in southern Brazil, using the Portuguese version of the Nasal Obstruction Symptom Evaluation (NOSE-p) and Rhinoplasty Outcome Evaluation (ROE), and also to evaluate the frequency of possible complications of this technique.

## Methods

### Study design and patients

We conducted a single-center prospective study with patients who had the indication for MES, from May 2016 to September 2020 at the Facial Plastic Surgery Clinic of Otolaryngology Department of the Hospital de Clinicas de Porto Alegre, a tertiary care university hospital in southern Brazil. Eligible patients were those aged ≥16-years old, with nasal obstruction and evidence of complex nasal septal deformities involving the L strut during physical examination which could not be eliminated with traditional septoplasty or rhinoplasty techniques. Some of these patients were also candidates to aesthetic primary rhinoseptoplasty. Exclusion criteria were deformities in which the keystone area was severely involved or in which the reminiscent cartilaginous nasal septum was displaced from the bone during the surgery and needed the traditional total dorsal reconstruction described by Gubish.^6^

### Data collection and outcomes

The primary outcome was the relative postoperative change in NOSE-p, a validated disease-specific quality-of-life questionnaire for assessing outcomes in nasal obstruction.^26^, ^27^ The variation in that scale goes from “0” which means “no nasal obstruction”, to “100”, which means “severe nasal obstruction”. Secondary outcome was the variation between “100” which means “total satisfaction”, and “0”, which means “major dissatisfaction” in ROE, a validated quality-of-life questionnaire for rhinoplasty patients.^28^

All patients were included in our protocol that consists of a brief questionnaire to provide demographic and baseline characteristics, detailed physical examination, nasal endoscopy, preoperative and postoperative standardized facial photography, and a questionnaire of associated complications in the postoperative period.

Also, at all medical appointments, patients were asked about allergic rhinitis symptoms and were classified according to Allergic Rhinitis and its Impact on Asthma (ARIA) guidelines^29^ as presenting (1) Intermittent or persistent or (2) Mild or moderate/severe symptoms. Starting at the 30-day postoperative visit, topical corticosteroids (budesonide 50 micrograms twice daily) were prescribed to patients presenting mild persistent or moderate/severe intermittent or persistent symptoms, according to ARIA guidelines. If mild persistent or moderate/severe intermittent or persistent symptoms were still present on the 60-day follow-up visit, high-dose topical corticosteroids were prescribed (budesonide 100 micrograms twice daily).

Those outcomes were assessed preoperatively and at 1-week and 1, 3, 6 and 12 months postoperatively and then annually, by trained investigators at the same day of clinical evaluation. Written informed consent was obtained from each patient before study enrollment.

### Surgical technique

#### Approach and nasal septal resection

**Fig. 1:**
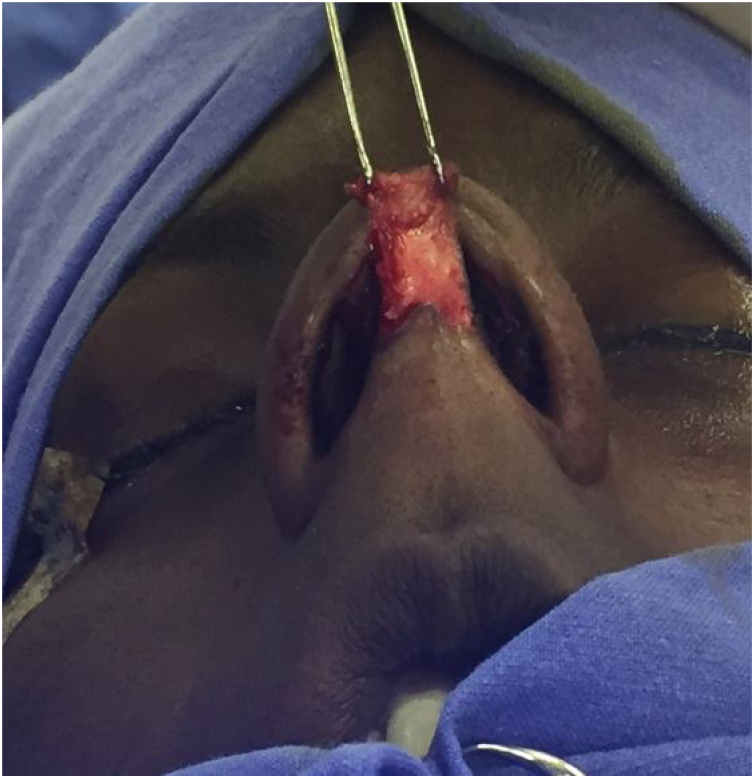
Septorhinoplasty the open approach (author’s archive).

**Fig. 2:**
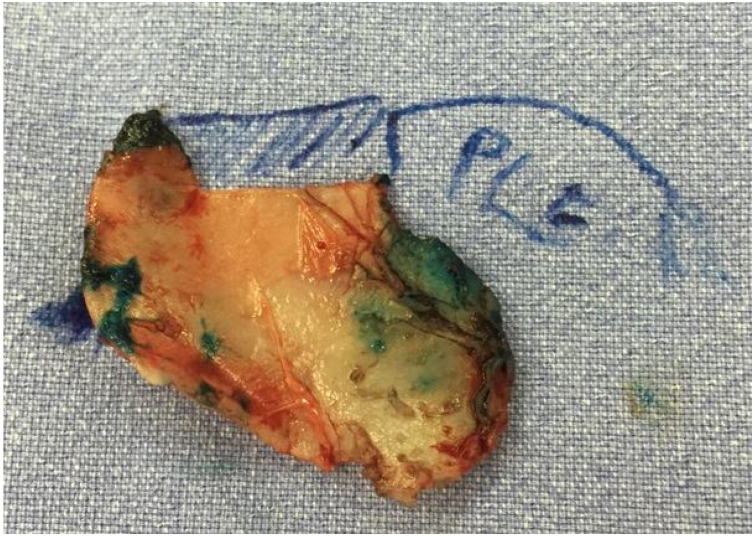
Diagram of original anatomy. PLE **‒** Perpendicular plate of ethmoid. Small strut represents the preserved dorsal nasal septal (author’s archive).

All patients undergo MES via an open approach (Fig. 1). Bilateral submucoperichondrial flaps were elevated and the upper lateral cartilages were released from the nasal septum. Then, if indicated, the cartilaginous dorsum hump was reduced to create a new keystone area. Like previously described by Most^4^ almost all cartilaginous septum was carefully removed, preserving at least 1.5 cm strut at the osteocartilaginous junction (Fig. 2). The main objectives were to remove all cartilaginous and bony deviated septum and to make sure that enough material for the neoseptum reconstruction was available.

#### Neoseptum reconstruction

**Fig. 3:**
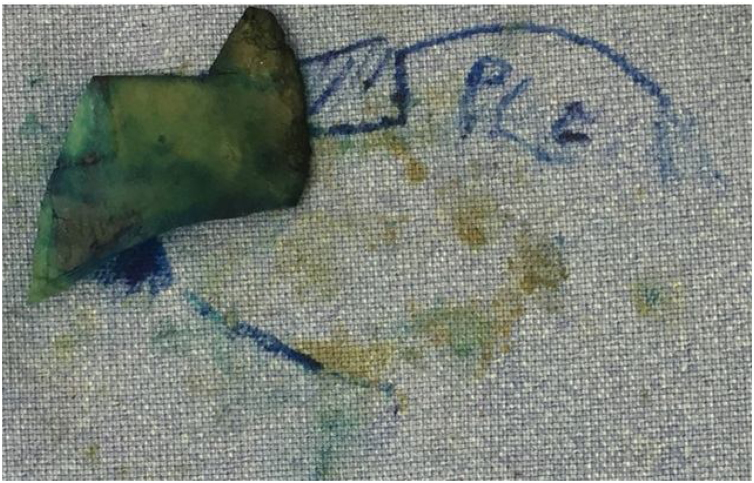
Septal reconstruction. The neoseptum is fabricated longer (cephalocaudal) than the native septum. So the neoseptum must connect the septal remancent (latero-lateral) and extend caudally to the anterior nasal spine thus extending caudally across the membranous septum to the caudal edge of the medial crura (author’s archive).

The neoseptum was rebuilt on the surgical table using the straightest portion so that its length should be able to connect the reminiscent native septum to the medial crura of inferior lateral cartilage and its height was attached to the desired nasal tip projection (Fig. 3). In all cases, the cartilage material was harvested by native septum and the use of ear or rib graft was not necessary. When needed, additional maneuvers were performed, like medial and lateral osteotomies or resection of bone hump.

#### Neoseptum fixation

The dorsal and caudal fixation of neoseptum were made by sutures. As described by Most,^4^ the neoseptum was carefully fixed with sutures latero-lateral to the most concave septal reminiscent in the K-stone area. In the present study sutures were performed with 5-0 Polydioxanone (PDS). After the dorsal stabilization of the neoseptum, unilateral or bilateral spreader grafts were attached with 5-0 polydioxanone if indicated (as in cases of nasal valve insufficiency, deviated nose or asymmetry of the middle third even after dorsal fixation). If a single spreader graft was indicated, it was placed along the dorsal reminiscent septum opposite to the side where the reconstructed septum was fixed.

**Fig. 4:**
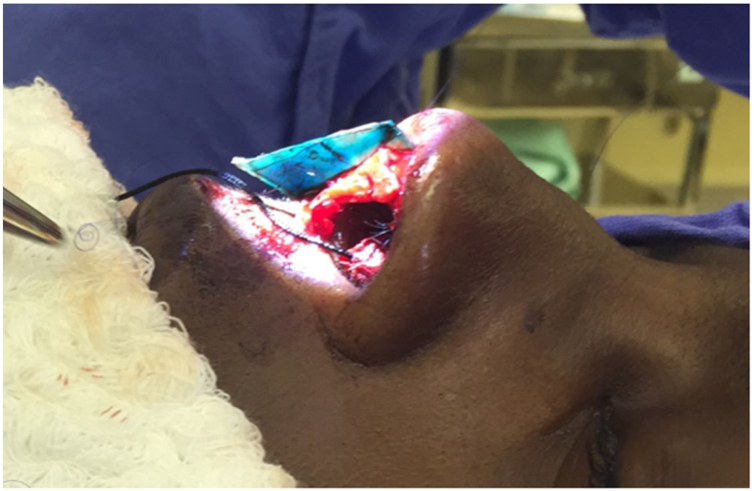
Neo septum fixation (author’s archive).

**Fig. 5:**
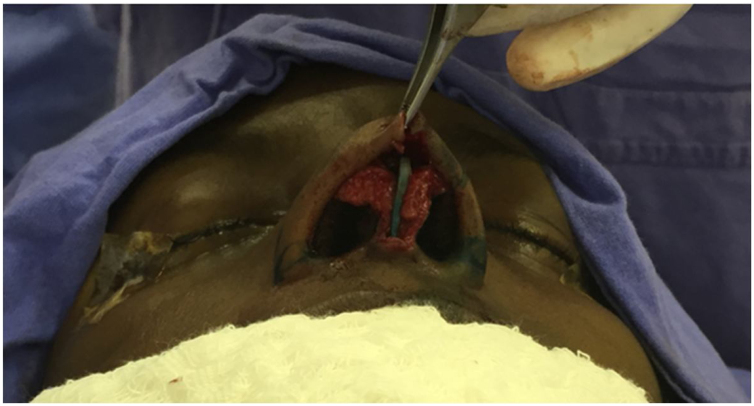
A tongue-in-groove stabilization with multiple PDS is performed between the medial crura and the caudal portion of the neoseptum (author’s archive).

The caudal fixation we performed as described by Wilson and Mobley,^25^ so the anterior to posterior (cephalocaudal) length of the reconstructed neoseptum needed to be long enough for the caudal edge of the neoseptum spans the membranous septum and ends caudally flush with the caudal edge of the medial crura of the inferior lateral cartilages. The caudal fixation of the neoseptum was made with multiple 5‒0 polydioxanone sutures in the medial crura of inferior lateral cartilage preserving the mucosa, as in a tongue-in-groove fashion (Fig. 4, Fig. 5). In the majority of cases lateral crural tensioning was realized. In some patients we performed interdomal or intradomal sutures if nasal tip refinement was planned. After closing the skin, plastic splints were placed along either side of the septum using 4.0 nylon sutures.

#### Postoperative management

All patients received perioperative intravenous antibiotics- Cefazolin sodium, 1–2 g; or clindamycin phosphate 900 mg, if the patient was allergic to penicillin. The antibiotics were administered according to the patient’s body weight. Antibiotic therapy was continued postoperatively with cephalexin, 500 mg 4 times per day; or clindamycin hydrochloride, 300 mg 3 times per day, for 7 days. The patient’s medical reevaluation occurred generally at the seventh postoperative day when the splints, the external curative and the columellar sutures were removed.

### Statistical analysis

Statistical analyses were performed using SPSS Statistics version 22 (IBM Corp., Armonk, NY). Normally distributed variables were described using means and standard Error (SEs) and were analyzed using 2-tailed paired t tests. Outcomes were described as changes in postoperative MES versus preoperative MES scores. A *p-*value < 0.05 was considered as a significant statistical difference.

## Results

Of the 31 patients submitted to the modified extracorporeal septorhinoplasty who were evaluated for entering the study, twenty-seven patients were included. Four were excluded due severe deformities in the keystone area and displacement of the cartilage from the bone on this area during the surgery and needed total dorsal reconstruction, as described by Gubish.

Mean age was 35.85 (±15.57) years, 12 (44%) were female and mean follow-up was 16.77 (1–36) months (Table 1).

**Table 1:** Baseline characteristic.

Characteristics	n (%) or mean (SD)
Sex (female)	12 (44)
Age, y	35.85 (± 15.57)
Caucasian	24 (89)
Postoperative follow-up (month)	16.77 (1−36)
Formal education, years	
8	13 (48)
9‒11	11 (41)
>12	3 (11)
Previous nasal trauma	7 (26)
Nasal symptoms	
Rhinorrhea	6 (22)
Nasal sneezing	11 (41)
Nasal itching	6 (22)
Allergic Rhinitis (AR)	
Intermitent	6 (22)
Persistent	9 (33)
Moderate/severe AR symptoms	24 (89)
Current use of topical nasal corticosteroid	12 (44)
Self-reported chronic disease	6 (22)

SD, Standard Deviation.

**Fig. 6:**
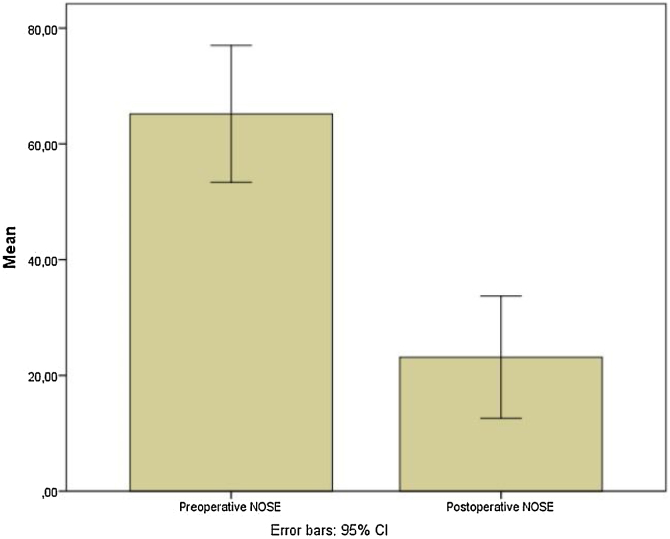
Box whisker plots of pre and postoperative Nasal Obstruction Symptom Evaluation-Portuguese scale of patients submitted to Modified Extracorporeal Septoplasty.

**Fig. 7:**
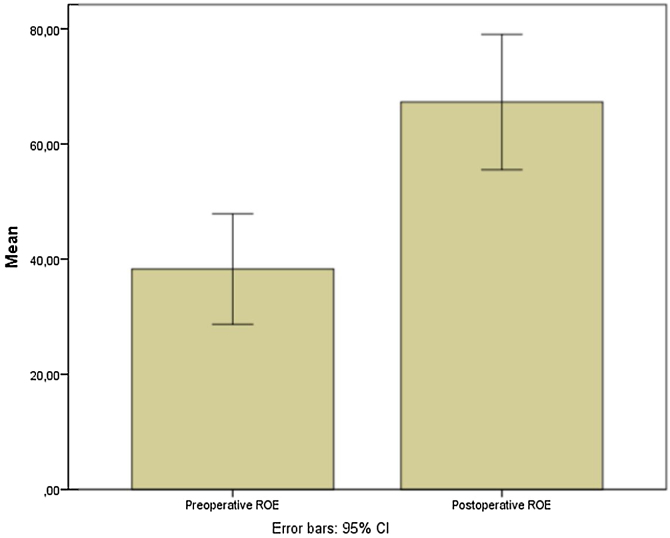
Box whisker plots of pre and postoperative ROE score of patients submitted to Modified Extracorporeal Septoplasty.

Preoperative and postoperative NOSE-p scale scores were 65.2 ± 29.9 and 23.5 ± 26.7, respectively (mean differences of 42.04; [95% CI 27.35–56.73]; *p* <  0.0001) (Fig. 6). Furthermore, pre and postoperative ROE scores were 38.3 ± 24.3 vs. 67.29 ± 29.7, respectively (mean differences of −29.02; [95% CI −40.5 to −17.5; *p* =  0.0001) (Fig. 7).

Residual septal deviation was verified in 2 patients (7.4%). Only 1 patient (3.7%) presented an asymptomatic synechia and a minor and asymptomatic septal perforation anteriorly in the septal mucosa. No severe epistaxis episodes were reported between consultation and no nasal packing was needed in any studied patient (Table 2, Table 3).

**Table 2:** Partial results of NOSE for each patient in the preoperative, 1-month postoperative, 3-month postoperative, 6-month postoperative and the scale results at the last consultation.

Patient	Treatment for allergic rhinitis	Follow-up (month)	Preoperative NOSE	Postoperative NOSE ‒ 1-month	Postoperative NOSE ‒ 3-months	Postoperative NOSE ‒ 6-months	NOSE at last appointment
1	YES	12	90.0	10	10	45	40.0
2	YES	24	35.0	20	25	55	30.0
3	NO	36	85.0	15	0	0	0.0
4	NO	12	100.0	‒	‒	‒	25.0
5	NO	36	40.0	80	10	10	0.0
6	YES	24	25.0	5	0	0	35.0
7	NO	2	60.0	12.50	‒	‒	0.0
8	NO	36	95.0	15	10	5	5.0
9	NO	24	100.0	60	10	0	0.0
10	YES	12	85.0	20	50	20	15.0
11	YES	27	80.0	25	35	30	70.0
12	NO	24	75.0	50	50	0	0.0
13	NO	3	75.0	–	25.0	‒	25.0
14	NO	12	65.0	15	0	5	0.0
15	NO	24	85.0	15	0	0	10.0
16	NO	8	85.0	60	0	0	0.0
17	YES	12	100.0	15	10	25	25.0
18	NO	12	65.0	5	0	5	35.0
19	NO	18	0.0	10	5	5	5.0
20	NO	12	0.0	25	20	10	10.0
21	NO	24	40.0	60	35	5	5.0
22	NO	12	70.0	5	0	0	0.0
23	YES	9	65.0	75	95	95	95.0
24	YES	9	95.0	75	75	70	75.0
25	NO	1	30.0	0	–	–	35.0
26	NO	6	30.0	10	0	10	10.0
27	NO	3	85	30	35	–	35

NOSE, Nasal Obstruction Symptom Evaluation.

**Table 3:** Partial results of ROE for each patient in the preoperative, 1-month postoperative, 3-month postoperative, 6-month postoperative and the scale results at the last consultation.

Patient	Follow-up (month)	Preoperative ROE	Postoperative ROE ‒ 1-month	Postoperative ROE ‒ 3-months	Postoperative ROE ‒ 6-months	ROE at last appointment
1	12	8.30	95.8	75	83.33	66.70
2	24	45.80	95.8	91.66	100	75.00
3	36	29.20	87.5	87.5	100	100.00
4	12	54.20	**‒**	**‒**	**‒**	37.50
5	36	41.70	58.3	91.2	75	75.00
6	24	33.30	70.8	66.7	75	41.70
7	2	12.50	0.00	**‒**	**‒**	0.00
8	36	33.30	95.8	95.8	95.8	95.80
9	24	50.00	83.3	83.3	95.8	95.80
10	12	41.70	54.2	58.3	75	66.70
11	27	25.00	54	54.2	58.3	37.50
12	24	45.80	58.3	87.5	100	100.00
13	3	4.20	**‒**	33.30	**‒**	33.30
14	12	95.80	79.2	87.5	87.5	87.50
15	24	45.80	95.8	87.5	100	70.83
16	8	45.80	58.3	62.5	62.5	62.50
17	12	70.80	41.7	91.7	100	100.00
18	12	8.30	95.8	95.8	100	83.30
19	18	45.80	83.3	83.3	79.16	79.20
20	12	16.70	54.2	83.3	83.3	79.20
21	24	62.50	70.83	79.2	100	100.00
22	12	33.30	75	87.5	87.5	91.70
23	9	41.70	33.3	33.3	66.7	4.20
24	9	8.30	50	70.3	54.2	25.00
25	1	0.00	62.5	**‒**	**‒**	62.50
26	6	91.70	91.7	95.8	87.5	87.50
27	3	41.7	70.8	70.8	**‒**	70.8

ROE, Rhinoplasty Outcome Evaluation.

## Discussion

Septoplasty is among the most frequently performed procedures in otolaryngology and facial plastic surgery.^8^ Standard in-situ septoplasty approaches are beneficial for most patients with mild to moderate middle or posterior septal deviations. Egmond et al. described in a randomized controlled trial a significant improvement in quality of life among patients who underwent septoplasty and turbinate surgery when compared with patients with isolated clinical treatment.^30^

However, the standard septoplasty techniques are not as effective for more severe deformities, especially if located in the anterocaudal septum, in the L-strut area.^24^ In order to effectively address those complex septal defects, the extracorporeal septoplasty technique was developed, and subsequently underwent modifications aiming to preserve the keystone area consequently reducing the risks of destabilization of the nasal dorsum and also modifying the anterior fixation to facilitate the surgical technique.

A statistically significant reduction in the postoperative NOSE score was reported in this study, a mean reduction of −42.04, which means improvement in nasal obstruction symptoms. Some studies also described similar results. Marangi et al., in a study with patients submitted to open and close access modified extracorporeal septoplasty, showed an improvement in NOSE score and objective nasal flow after 6-months of postoperative follow up in both groups.^31^ Likewise, Persichetti et al. described in a prospective study of patients with 5-years of MES postoperative follow up, a statistically significant improvement in inspiratory flow and obstructive symptoms across the entire cohort by means of rhinomanometric analysis (*p* = 0.0122) and NOSE questionnaire responses (*p* < 0.0001).^32^

Prospective studies worldwide concerning surgical outcomes in extracorporeal septoplasty and its modifications with good methodology are few. In a meta-analysis of outcomes of extracorporeal septoplasty and its modifications in treatment of severe L-strut septal deviation,^24^ analysis of surgical outcomes could only be performed using 5 studies,^4^, ^33^, ^34^, ^35^, ^36^ all modified ECS techniques reporting NOSE outcomes. The others analyzed studies could not be included because of variable methods to report results. Additionally, less than half of the studies (14 of 31 [45.2%]) were considered to be of good methodology according to the Guidance for Assessing the Quality of Before-After (Pre-Post) Studies with No Control Group. This meta-analysis of these 5 studies showed a change in total NOSE score of −60.0 (95% CI −67.8 to −52.2) points, indicating both a clinically and statistically significant improvement of nasal obstruction.

Only one of the studies above^34^ was done with Brazilian patients, in a preliminary report with 10 individuals with 60-days follow-up. The technique was restricted to anterior septal deviations through a surgical maneuver whereby the entire deviated portion was removed and a straight cartilage segment was placed between the medial crura of the alar cartilages, through a retrograde approach, to support the nasal tip. In the current Brazilian study, we followed a cohort with more patients and a longer follow up (16.77-months, on average), with evidence of complex nasal septal deformities involving the L strut during physical examination, that is, with broader indications.

In the current study, good aesthetical results were observed too, once an increase in postoperative ROE score was observerved: pre and postoperative ROE scores were 38.3 ± 24.3 vs. 67.29 ± 29.7, respectively. Persichetti et al. described in a cohort of one hundred and twenty patients submitted MES that all patients were subjectively satisfied with the aesthetic postoperative outcome.^32^

Regarding the possible complications of MES of the 11 studies on modified extracorporeal septoplasty analyzed in a meta-analysis,^24^ there were 6 infections (0.8%), no bleeding events, 12 nasal dorsal irregularities (1.7%), 6 other cosmetic complications (0.86%) and 7 other functional complications (1.0%). Unsal et al. described reduction in nasal tip projection and rotation among patients submitted to isolated modified extracorporeal septoplasty without concomitant rhinoplasty.^37^ In the current study, we reported a low index of complications after MES: residual septal deviation was verified in 2 patients (7.4%), 1 patient (3.7%) presented an asymptomatic synechia and a minor and asymptomatic septal perforation anteriorly in the septal mucosa.

## Conclusion

This is the longest prospective study with Brazilian individuals presenting complex septal deviations treated with MES. In the present study, most of the patients submitted to modified extracorporeal septoplasty had a significant improvement in quality-of-life scores of nasal obstructions, with good aesthetical outcomes and low indices of postoperative complications.

## Authors’ contributions

R.O.M and M.L: Conceptualization, Methodology, Data curation, Writing-Original draft preparation. O.E.S, E.P.F: Methodology, Writing-Original draft preparation. O.A.G.D, L.F.S and B.L.B: Conceptualization, Supervision, Writing- Reviewing and Editing.

## Consent for publication

All authors have reviewed the final version of the manuscript and agree with the publication of the results presented.

## Funding

This research received no external funding.

## Conflicts of interest

The authors declare no conflicts of interest.
